# High incidence of functional ion-channel abnormalities in a consecutive Long QT cohort with novel missense genetic variants of unknown significance

**DOI:** 10.1038/srep10009

**Published:** 2015-06-12

**Authors:** Annette Buur Steffensen, Marwan M. Refaat, Jens-Peter David, Amer Mujezinovic, Kirstine Calloe, Julianne Wojciak, Robert L. Nussbaum, Melvin M. Scheinman, Nicole Schmitt

**Affiliations:** 1 Danish National Research Foundation Centre for Cardiac Arrhythmia, Department of Biomedical Sciences, University of Copenhagen, Copenhagen, Denmark; 2 Department of Internal Medicine, Division of Cardiology, American University of Beirut Medical Center, Beirut, Lebanon; 3 Department of Medicine and; 4 Institute for Human Genetics, University of California San Francisco, California, U.S.A

## Abstract

The Long QT syndrome (LQTS) is a disorder characterized by a prolongation of the QT interval and a propensity to ventricular tachyarrhythmias, which may lead to syncope, cardiac arrest, or sudden death. Our objective was to (1) determine the incidence of variants with unknown significance (VUS) in a cohort of consecutive LQTS patients and (2) to determine the percentage of those with novel missense VUS that have demonstrable functional channel abnormalities from a single referral center. We performed genetic screening of candidate genes in 39 probands with a diagnosis of LQTS to identify mutations and variants. Seven variants of unknown significance were identified, six were missense variants and one was a splice site variant. We investigated the six novel missense VUS in five patients; three missense variants in *KCNQ1* (L236R, W379R, Y522S) and three missense variants in *KCNH2* (R35W, S620G, V491I). We employed two-electrode voltage-clamp experiments in *Xenopus laevis* oocytes and confocal imaging to characterize the novel missense mutations functionally. We revealed electrophysiological and trafficking loss-of-function phenotypes. This report emphasizes the frequency of adverse channel function in patients with LQTS and the importance of heterologous studies to define channel function.

The Long QT Syndrome (LQTS) is an inherited cardiac arrhythmic syndrome with a prevalence of 1:2500^1^. The diagnostic criteria include prolongation of the QT interval on the electrocardiogram (ECG), syncope, polymorphic tachycardia of the *torsade de pointes* (TdP) type, and family history^2^. LQTS is caused by mutations in genes encoding ion channels or proteins that modify ion channel function. At present, thirteen genes have been associated with the disease, all of them encoding ion channels or proteins that modify ion channel function (for review see^3^). Most mutations identified so far reside in the genes *KCNQ1*, *KCNH2*, and *SCN5A* encoding the voltage-gated potassium channels K_V_7.1 and K_V_11.1 and the cardiac sodium channel Na_V_1.5, respectively. K_V_7.1 co-assembles with the beta-subunit KCNE1, a single transmembrane peptide, to form the I_Ks_ channel complex^4^,^5^. K_V_11.1 is the molecular entity of I_Kr_^6^. Together, these delayed rectifier potassium currents are crucial for the repolarization of the cardiac action potential and mutations in the underlying genes account for 80-90% of all hereditary LQTS^7^,^8^.

To date, approximately 20–25% of individuals with clinical features of LQTS have no identified genetic abnormality^3^. A number of challenges exist in clinical genetic testing for LQTS. First, the field is rapidly evolving and reveals new variants whose pathogenicity is often difficult to assess. Furthermore, different clinical testing laboratories have used and continue to use different categories of pathogenicity assessment, despite the proposals by the American College of Medical Genetics, that laboratories use a uniform scale for classifying mutations^9^. Even if a uniform classification scheme is used, each laboratory has its own criteria for deciding which factors should be considered and how different factors should be weighted when deciding into which category of pathogenicity any one variant should be assigned. These factors include whether (1) the variant has been seen before in affected unrelated individuals, (2) the variant is found in presumably unaffected reference populations and with what frequency, (3) there is evidence for co-segregation of the variant and long QT syndrome in one or more families, (4) there is *in vitro* functional evidence that the variant disrupts protein function, and (5) *in silico* analyses predict an impact of the variant depending on the domain of the protein in which it is located and whether, if a missense mutation, the affected residue is conserved or whether a different variant affecting that same residue shows some evidence for being disease-causing. Finally, only a minority of all variants and their pathogenicity assessments are in the public domain, either in peer-reviewed literature or in public databases, making it difficult for any single testing laboratory to use everything that has been discovered about the variant by other clinical testing laboratories when making its assessment of what to the laboratory appears to be a novel variant.

The purpose of our study was to (1) determine the incidence of VUS in a cohort of consecutive patients seen in our clinic for evaluation of LQTS and (2) determine the percentage of those with novel missense VUS that have demonstrable functional channel abnormalities from a single referral center.

## Results

### Mutation screening

Variants were classified when reported based on the variant interpretation standards recommended by the American College of Medical Genetics^9^. Deleterious mutations were identified in 17 (44%) patients, 11 (28%) patients had genetic VUS, and no alterations were identified in 11 (28%) (Supplemental Table S1). Cases were included in this experimental study based on (1) novelty of a missense VUS, (2) family segregation, or (3) presence of at-risk family members to benefit from testing. Specifically, out of the 39 probands who underwent clinical testing, five patients had novel missense VUS and were chosen for functional assessment. Four of the probands were chosen because the clinical lab classification most closely conformed to either a novel missense VUS or a novel missense VUS, likely disease causing, category^9^. The fifth patient was chosen because two novel missense VUS were identified.

### Clinical characterization

Proband-1 was diagnosed at age 37 after cardiac arrest following severe vomiting due to motion sickness while on a cruise. She had QT prolongation and documented TdP when hypokalemic. She was diagnosed with LQTS and an ICD was implanted. Another episode of nausea and vomiting led to appropriate multiple shocks for TdP approximately 2 years ago. Her arrhythmias were again accompanied by low potassium levels. She was treated with overdrive atrial pacing, augmentation of her beta blocker therapy and by repletion of K^+^ with amelioration of symptoms. There was a family history of sudden death (SD) (Supplemental Figure S1). A paternal uncle who was reported to be very healthy died unexpectedly in his sleep in his 40s. Another paternal uncle died in his 20s in a single vehicle accident, this accident was attributed to drunk driving. Her son reportedly had an ECG with a QT interval of 467 ms, but the report was not available for review. Proband-1 carries the novel variant K_V_11.1–R35W. This residue resides in the so-called Per-Arnt-Sim (PAS) domain that has an impact on deactivation properties of the channel^10^.

Proband-2 had syncopal episodes with auditory stimuli since childhood and was misdiagnosed with a seizure disorder. At age 38, she had cardiac work-up that revealed prolonged QT intervals ranging from 493–515 ms. She had documented TdP on Holter recordings, and an ICD was implanted. The proband’s father is healthy with a normal QTc interval. Proband-2 carries K_V_11.1-S620G. The amino acid residue resides in transmembrane segment S6, is conserved across species and plays a role in channel inactivation^11^,^12^,^13^,^14^. The proband’s mother, father, and sister all tested negative for this variant suggesting it is a *de novo* mutation.

**Figure 1:**
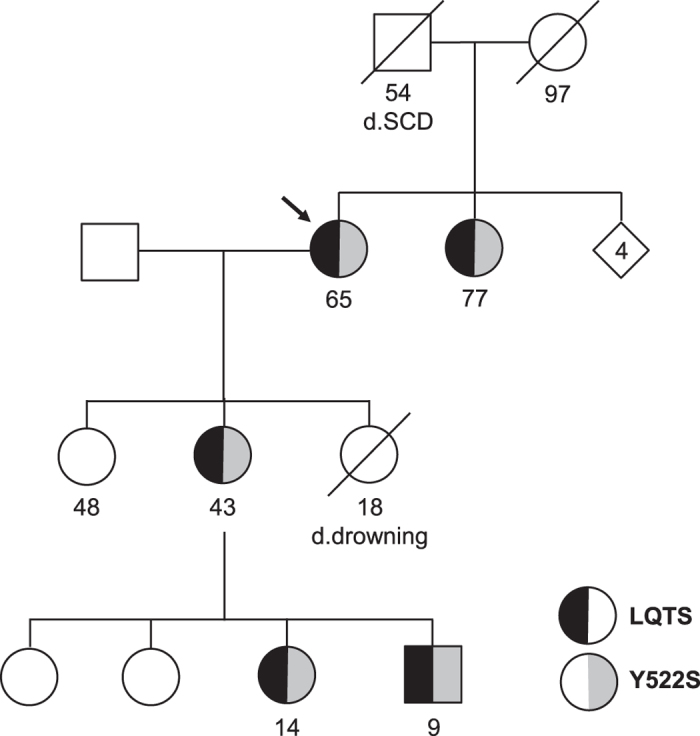
Clinical and genetic information of Proband-3 carrying mutation K_V_7.1 Y522S. Circles: female, Squares: male. Arrow indicates Proband-3.

Proband-3 is a 65 years old woman with a history of syncope around age 57 during fever. Multiple ECGs were reportedly taken over the years, and five out of seven showed QTc prolongation. The longest noted QTc was 510 ms. There is an extensive family history of LQTS and SD (Fig. 1). The proband’s daughter drowned at age 18 in 1983 despite being an experienced swimmer. Her granddaughter was diagnosed with LQTS at age 7 based on syncopal episodes. She was treated with β-blockers and received an ICD. Her grandson was diagnosed with LQTS at age 9 and is managed with β-blockers. The proband’s daughter was then evaluated, diagnosed with LQTS at age 36 and received an ICD. Her sister was diagnosed with LQTS at age 72 after a syncopal episode. She received an ICD and had one appropriate shock in 2011 at age 76. Her father died suddenly of a suspected stroke at age 54. Proband-3 carries the novel variant K_V_7.1-Y522S which resides in a homologous region of the K_V_7.1 C-terminus that has been implicated in subunit assembly^15^. Her daughter, granddaughter, grandson, and sister all tested positive for this variant.

Proband-4 presented with syncope while swimming at age 11. She regained consciousness, was found to have a prolonged QTc on multiple ECGs and eventually received an ICD. Family history is significant for a paternal uncle who died in his 30s thought to be due to a drug overdose but there is limited information. The parents and 10 years old sister reportedly have had normal baseline ECGs in the past. Proband-4 carries the novel variant K_V_7.1–L236R that resides in S4 and introduces a positive charge into the voltage-sensor domain (Supplemental Figure S2).

Proband-5 presented with a history of palpitations and QT prolongation on multiple ECGs (482–520 ms). She has chronic obstructive pulmonary disease and has had palpitations for about 12–15 years. Some episodes of feeling faint were reported but these did not occur with palpitations. A nephew drowned at age 24 under the influence of alcohol. A cousin who reportedly had epilepsy died at the age 14. She carries a novel variant K_V_7.1–W379R that resides in helix A of the channel protein. This region is involved in binding of the calcium sensor calmodulin, and a role in trafficking has been suggested^16^,^17^. In addition, the proband carries a novel variant in K_V_11.1, V491I (Supplemental Figure S3).

All carriers were female and heterozygous for the variants. The variants identified in this study are listed in Supplemental Table S1; a schematic overview of the positions in the channel proteins is depicted in Supplemental Figure S4. All novel variants affect residues conserved across species (Supplemental Figure S4) and are absent in the 1000 Genomes and Exome Variant Server databases.

### Mutant K_V_11.1 channels R35W and S620G display loss-of-function

To investigate if the novel K_V_11.1 variants could explain the LQTS phenotype observed in the probands, we expressed wild-type (WT) or mutant in *Xenopus laevis* oocytes and performed two-electrode voltage-clamp (TEVC) experiments. K_V_11.1 currents were elicited by applying depolarizing voltage-steps in 10 mV increments from −80 to 40 mV for 1 s. Tail currents were recorded after stepping to −60 mV.

**Figure 2:**
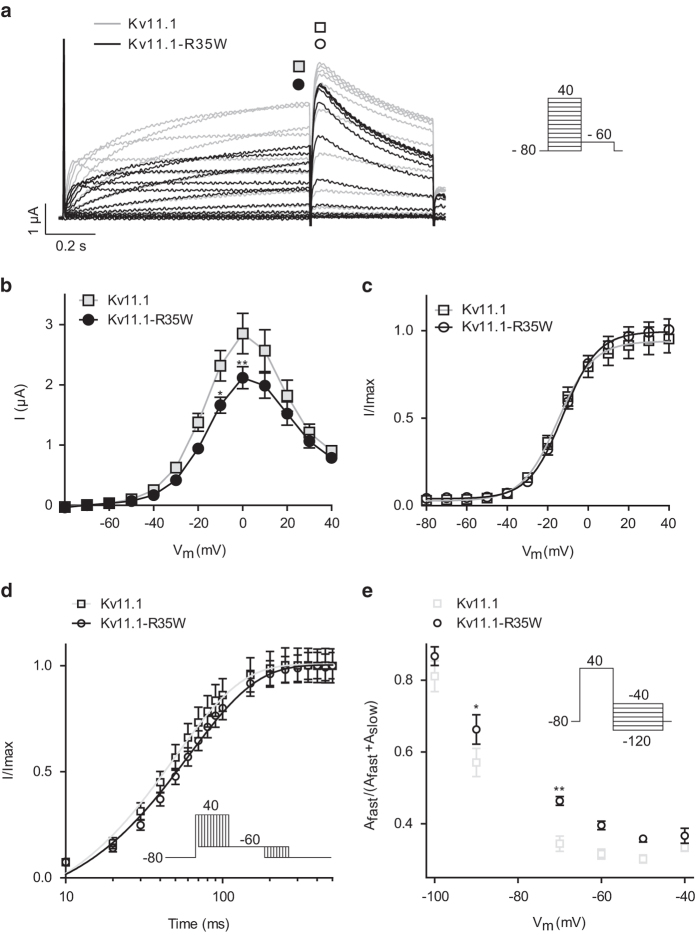
Characterization of K_V_11.1–R35W. **a**: Representative current traces recorded from *X. laevis* oocytes injected with K_V_11.1 or K_V_11.1–R35W cRNA. The current step protocol is shown as inset. **b**: Currents at the end the steps (points indicated by filled squares and circles in **a**) were plotted as a function of voltage to depict the current-voltage (IV) relationship for K_V_11.1 (at 0 mV; 2.9 ± 0.3 μA, n = 5) and K_V_11.1–R35W (at 0 mV; 2.1 ± 0.2 μA, n = 17). **c**: Normalized tail current as measured from peak current indicated by open squares and circles in A resulted in the voltage-dependent activation. For K_V_11.1 the half-maximal activation voltage (V_1/2_) was −15.0 ± 0.6 mV (n = 14) and for K_V_11.1–R35W: V_1/2_ = −12.2 ± 0.3 mV (n = 15). **d**: Activation kinetics were addressed by an envelope of tails protocol (inset). Data were normalized to the maximum amplitude of the tail current and plotted on a log time scale for K_V_11.1 (49.5 ± 2.3 ms, n = 15) and K_V_11.1–R35W (61.3 ± 2.3 ms, n = 18). **e**: The kinetics of deactivation (protocol shown as inset) were only altered for the relative contribution of the fast component of K_V_11.1 (at −70 mV; 0.34 ± 0.02, n = 13) compared with K_V_11.1–R35W (at −70 mV; 0.46 ± 0.01, n = 16). *P < 0.05, **P < 0.01.

Representative recordings of WT and K_V_11.1–R35W are shown in Fig. 2a. The current/voltage relationship (Fig. 2b) recorded at the end of the steady-state step was characteristically bell-shaped due to inactivation of the K_V_11.1 current^6^. Current levels at 0 mV were significantly reduced by approximately 26% for the mutant channels compared with WT, yet the voltage-dependent activation remained unchanged (Fig. 2c). The pseudo-time constant of activation was significantly higher at + 40 mV for K_V_11.1–R35W (Fig. 2d) resulting in slower activation. Deactivation was altered where the relative contribution of the fast component (A_fast_) was more important at −90 and −70 mV (Fig. 2e) for K_V_11.1–R35W than WT. None of the other kinetic parameters were changed (data not shown). As the patient’s arrhythmic episodes were suspected to be provoked by low potassium levels, we repeated the studies using low extracellular potassium concentration (Supplemental Figure S5). Lowering extracellular K^+^ from 4 to 1 mM led to a reduction of the steady state current at 0 mV by 54 ± 3% (reaching 1.2 ± 0.1 μA) for the WT channel in agreement with earlier reports^6^, whereas the mutant K_V_11.1-R35W was reduced by 40 ± 1% (at 0 mV; 1.0 ± 0.1 μA). The voltage-dependence of activation was not affected (Supplemental Fig.S5).

**Figure 3:**
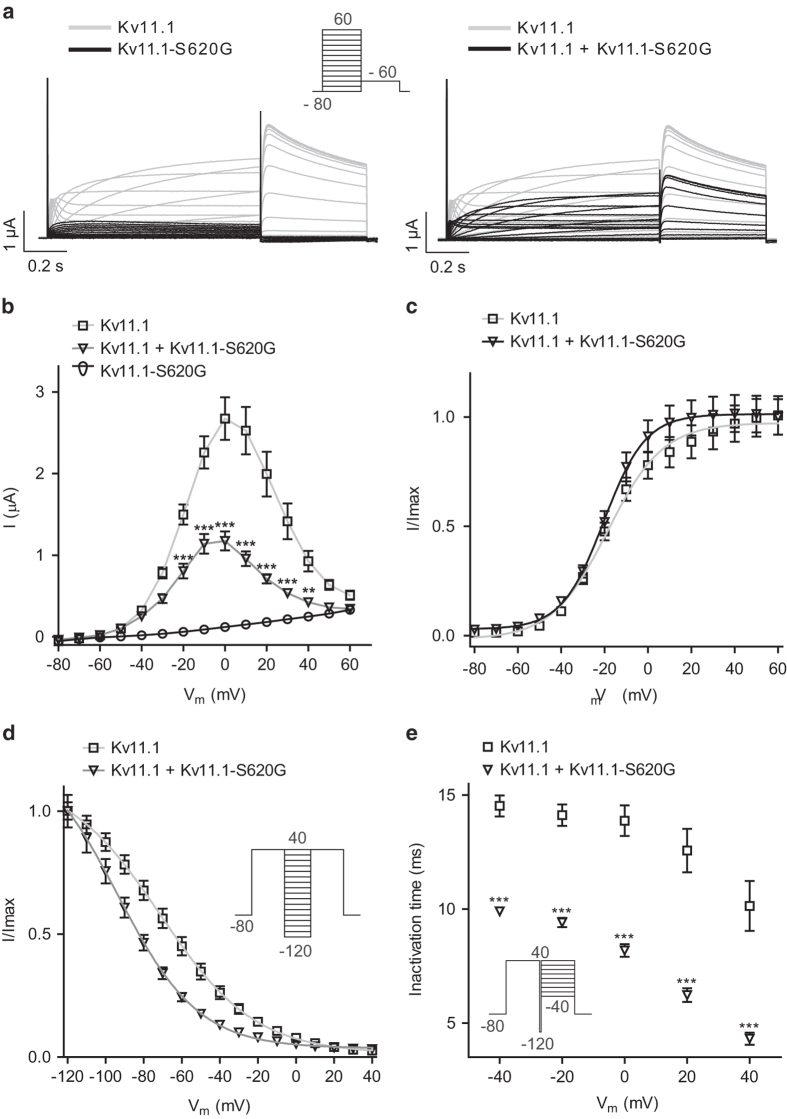
Characterization of K_V_11.1–S620G. **a**: *X. laevis* oocytes were injected with K_V_11.1, K_V_11.1-S620G, or K_V_11.1 + K_V_11.1–S620G (1:1 molar ratio) cRNA and currents recorded by TEVC. The protocol is shown as inset. **b**: I/V relationship for K_V_11.1 (at 0 mV; 2.7 ± 0.3 μA, n = 19), hetero (at 0 mV; 1.2 ± 0.1 μA, n = 24) and K_V_11.1-S620G (at 0 mV; 1.2 ± 0.1 μA, n = 11). **c**: Normalized tail current as measured from peak current resulted in the voltage-dependent activation. For K_V_11.1 the half-maximal activation voltage (V_1/2_) was −18.9 ± 0.7 mV (n = 18) and for hetero: V_1/2_ = −20.0 ± 0.4 mV (n = 24). **d**: Voltage-dependent recovery from inactivation was determined from the protocol in the inset where a Boltzmann function was fit to the normalized peak current of K_V_11.1 (V_1/2_ = −69.8 ± 2.7 mV, n = 17) and of hetero (V_1/2_ = −93.4 ± 1.8 mV, n = 17) E: Inactivation time was determined from a mono-exponential fit to the tail currents. **P < 0.01, ***P < 0.001

Expression of K_V_11.1–S620G showed complete loss-of-function for the mutant channel (Fig. 3). To investigate whether in the heterozygous state observed in the patient the mutant channel subunits were able to affect WT subunits, we co-expressed WT and mutant channels in a 1:1 molar ratio. Representative current traces are shown in Fig. 3a, current-voltage relationships in Fig. 3b. We observed a reduction of approximately 56% of the steady state current at 0 mV compared to WT channels, yet no alteration of the voltage-dependent activation (Fig. 3c). We addressed the voltage-dependent recovery from inactivation by a step to + 40 mV, followed by brief (10 ms) hyperpolarization from + 40 to −120 mV in 10 mV decrements and returning to + 40 mV. The voltage-dependent recovery from inactivation was significantly shifted in the negative direction by approximately 22 mV for hetero compared to WT (Fig. 3d). Furthermore, the kinetics of inactivation were investigated by a step to + 40 mV, then a brief (10 ms) hyperpolarizing step to −120 mV, followed by a final step to potentials ranging from + 40 to −40 mV in 10 mV increments. Inactivation time-constants were evaluated from mono-exponential fits to the tail currents and plotted against the applied voltage. This revealed that the decreased current amplitude was due to significantly faster inactivation kinetics already at −40 mV (Fig. 3e).

K_V_11.1-V491I behaved similarly to WT channels (data not shown).

**Figure 4:**
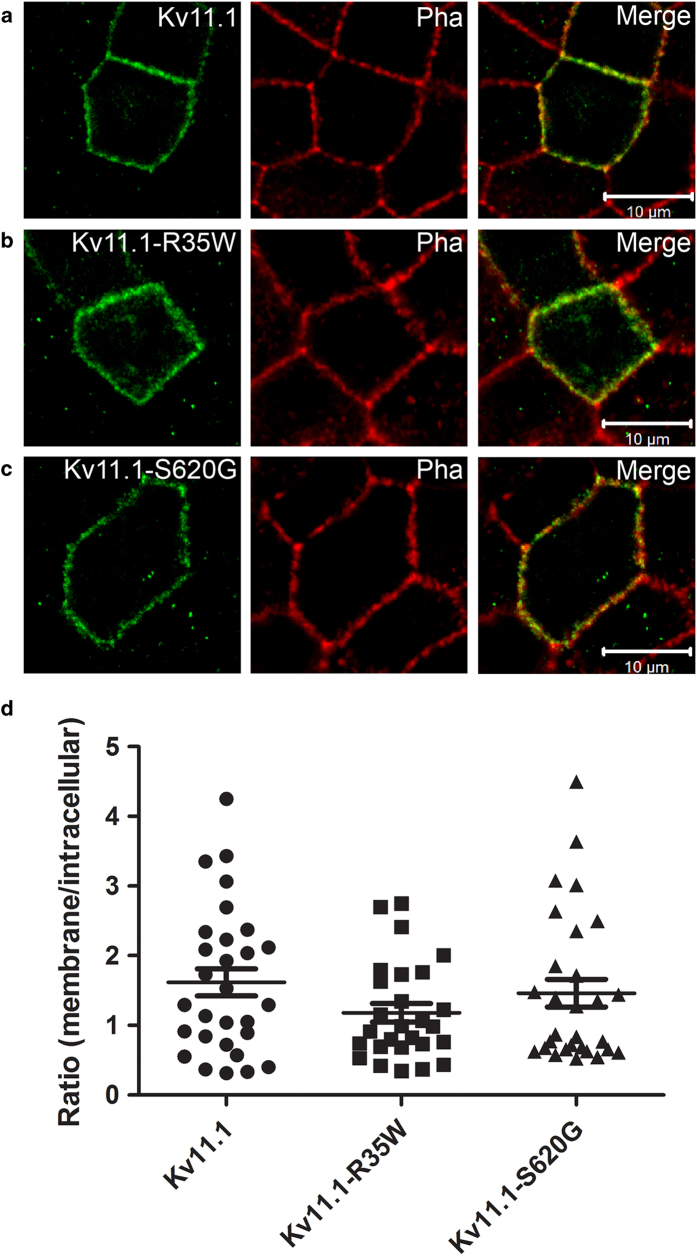
Subcellular localization of K_V_11.1 and mutants. MDCK cells were transiently transfected with K_V_11.1, K_V_11.1–R35W, or K_V_11.1–S620G and grown to confluency. Using a specific K_V_11.1 antibody the localization of the WT and the MUT channels were visualized with confocal microscopy. Phalloidin (Pha) was used as a membrane marker as it stains the F-actin just underneath the cell membrane. (**a**-**c**) All subunits were found capable of trafficking to the cell membrane in some cells. Merged pictures are shown in right panels. (**d**) Quantification compare the number of channels in the membrane to the number of channels trapped in intracellular compartments (most likely ER) for WT and mutants (*p* = 0.23, n = 27-29 cells for each situation).

We further assessed whether the functional phenotypes observed in TEVC experiments were due to changes in subcellular localization of the mutants using the epithelial Madin-Darby Canine Kidney (MDCK) cell line^18^. Phalloidin, which stains the F-actin located beneath the plasma membrane in MDCK cells, was used as surface membrane marker. Similar to WT, the mutant proteins K_V_11.1-R35W and K_V_11.1-S620G were mainly located in the membrane (Fig. 4a-c). We observed a high degree of cell-to-cell variability independent of the cell batch (Fig. 4d). We conclude that the loss-of-function phenotypes in electrophysiological experiments are not due to impaired trafficking.

### Mutant K_V_7.1 channels L236R, W379R, and Y522C display loss-of-function

**Figure 5:**
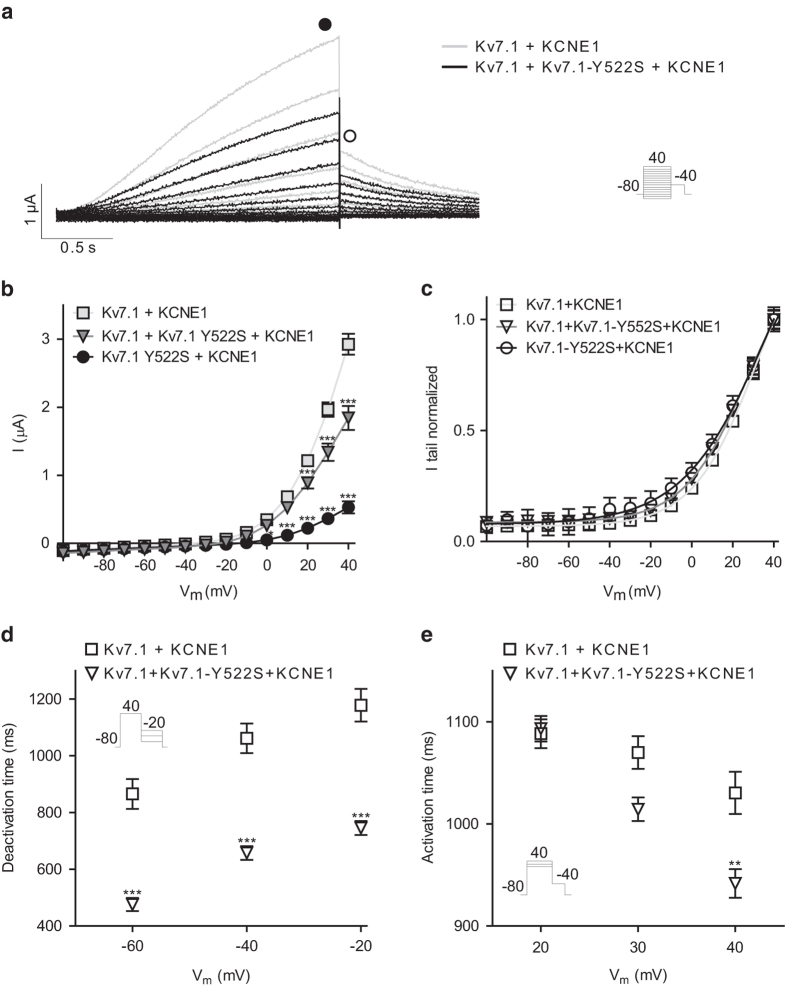
Characterization of K_V_7.1–Y522S. K_V_7.1 or K_V_7.1–Y552S were expressed with KCNE1 in a 1:1 molar ratio in *X. laevis* oocytes. **a**: Representative recordings. The voltage protocol is shown as inset. **b**: IV relationship for K_V_7.1 measured from points indicated with the filled square in **A** (at 40 mV; 2.9 ± 0.2 μA, n = 36), K_V_7.1–Y522S (at 40 mV; 0.5 ± 0.1 μA, n = 12), and hetero (at 40 mV; 1.8 ± 0.2 μA, n = 27). **c**: Voltage-dependent activation as measured peak tail current indicated by the open square in **A**. For K_V_7.1, the half-maximal activation voltage (V_1/2_) was 31.1 ± 0.7 mV; for K_V_7.1–Y522S: V_1/2_ = 38.4 ± 4.4 mV, and hetero: V_1/2_ = 28.6 ± 1.1 mV. **d**: Deactivation time constants were obtained by fitting a mono-exponential function to the tail-currents. The protocol is shown as inset. **e**: Activation time, determined as time-to-half maximal current at + 20, 30 or 40 mV. *P < 0.05, **P < 0.01, ***P < 0.001.

To mimic the native I_Ks_ current, we co-expressed K_V_7.1 and KCNE1. Co-expression of WT subunits gave rise to slowly activating and deactivating potassium currents and no inactivation when activated by a voltage-step protocol (10 mV increments, 2-s pulses) from a holding potential of −80 mV to + 40 mV. Representative recordings are shown in Fig. 5a.

Expression of K_V_7.1–Y522S showed significantly decreased current amplitudes at all activating potentials (Fig. 5b). Also co-expression of the mutant channel with WT resulted in decreased steady-state current amplitude with an approximately 37% reduction at + 40 mV suggesting a loss-of-function in I_Ks_ (Fig. 5b). Tail current analysis showed that the loss-of-function may at least partly be explained by a depolarizing shift of the voltage-dependent activation (Fig. 5c) when the mutant was expressed alone (P = 0.004), yet not reaching significance in the heterozygous state (P = 0.05). It should be noted that V_½_ values for the normalized voltage-dependent activation of K_V_7.1 WT and mutants in the presence of KCNE1 are only estimates since the currents do not reach saturation. To further investigate the effects of K_V_7.1–Y522S in the heterozygous situation, we analyzed the kinetic parameters. The kinetics of deactivation were determined by recording tail currents at potentials ranging from −60 to −20 mV in 20 mV increments after an activating step to + 40 mV. The best fit to the current traces of deactivation was a mono-exponential function. Figure 5d shows that expression of K_V_7.1 + K_V_7.1–Y522S leads to a significantly faster deactivation. Finally, we elicited currents at + 20, + 30 and + 40 mV followed by a tail step to −40 mV and determined time to half maximal current (t_½_) of activation. We found that the activation time was faster for the mutated channel co-expressed with the WT compared to WT alone at + 40 mV (Fig. 5e). The significantly faster deactivation kinetics and the depolarizing shift of the voltage-dependent activation suggest a loss-of-function mutation.

**Figure 6:**
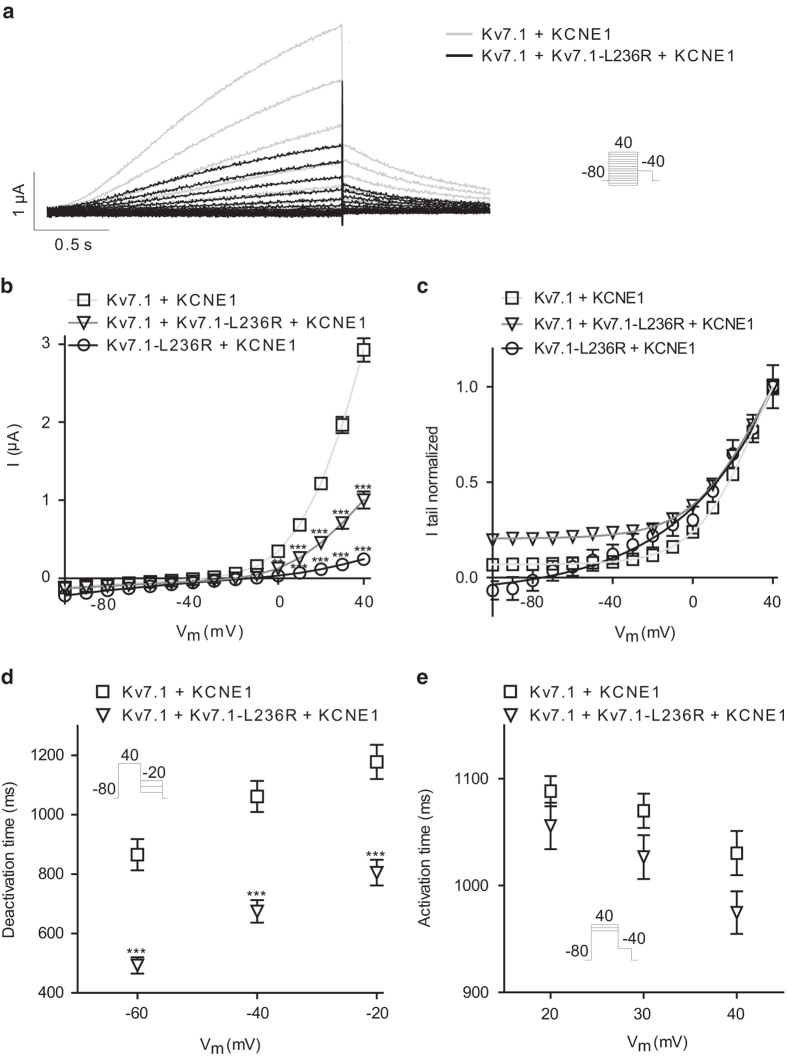
Characterization of K_V_7.1–L236R. K_V_7.1 or K_V_7.1–L236R expressed with KCNE1 in a 1:1 molar ratio in *X. laevis* oocytes. **a**: Representative recordings. The voltage protocol is shown as inset. **b**: IV relationship for K_V_7.1 (at 40 mV; 2.9 ± 0.2 μA, n = 44), K_V_7.1–L236R (at 40 mV; 0.2 ± 0.03 μA, n = 12), and hetero (at 40 mV; 1.0 ± 0.1 μA, n = 34). **c**: Voltage-dependent activation. For K_V_7.1, the half-maximal activation voltage (V_1/2_) was 31.1 ± 0.7 mV; for K_V_7.1–L236R: V_1/2_ = 85.4 ± 11.7 mV, and hetero: V_1/2_ = 31.2 ± 1.7 mV. **d**: Deactivation time constants were obtained by fitting mono-exponential functions to the tail-currents. **e**: Activation time, determined as the time to half maximal current at + 20, 30 or 40  mV. **P < 0.01, ***P < 0.001.

Decreased current amplitudes were also found for K_V_7.1–L236R (Fig. 6a). Mimicking the heterozygous state led to approximately 66% reduction of the steady state current at + 40 mV indicating a dominant-negative effect (Fig. 6b). Voltage-dependent activation of the channels was determined by fitting Boltzmann functions to normalized peak tail-currents (Fig. 6c). For WT, the half-maximal activation voltage (V_1/2_) was 31.1 ± 0.7 mV; for K_V_7.1–L236R: V_1/2_ = 85.4 ± 11.7 mV, and hetero: V_1/2_ = 31.2 ± 1.7 mV. In addition to the depolarizing shift of the voltage-dependent activation for the mutant expressed alone (Fig. 6c), we found significantly faster kinetics of deactivation (Fig. 6d). The time constant of activation was unaltered (Fig. 6e).

K_V_7.1–W379R showed a loss-of-function compared to WT channels with an approximately 92% reduction of the steady state current at + 40 mV for K_V_7.1–W379R alone and approximately 76% reduction for the heterozygous state which can at least partly be explained by a depolarizing shift of the voltage-dependent activation (Supplemental Figure S6).

**Figure 7:**
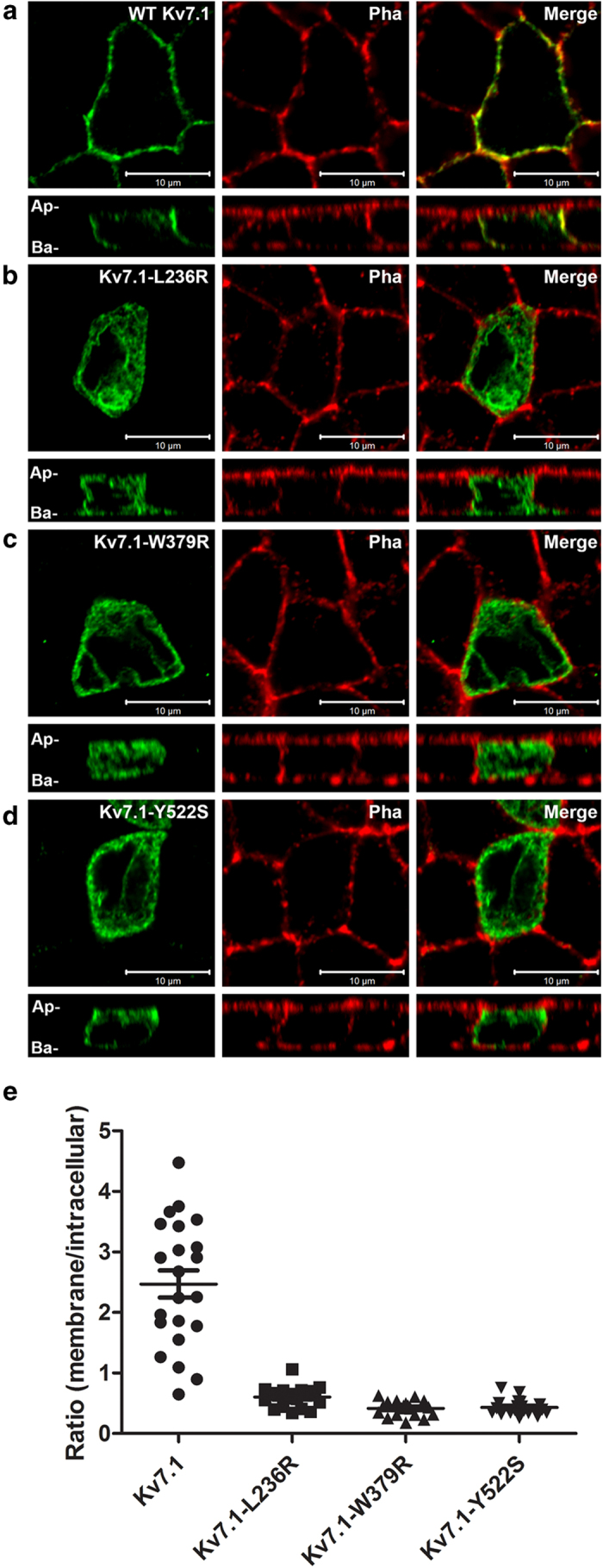
Subcellular localization of K_V_7.1 and mutants. Horizontal and vertical confocal images of polarized MDCK cells transiently expressing the K_V_7.1–WT or MUT as indicated and labeled with antibodies against K_V_7.1 (left panels) and Phalloidin (middle panels). Merged pictures are shown in right panels. Ap; apical; Ba, basolateral. Representative pictures from three independent experiments are shown. Quantification of ratio between channels in the membrane versus channels trapped in intracellular compartments (most likely ER) for WT and mutants (*p* < 0.0001, n = 18−22 cells each situation).

Addressing the subcellular localization of the mutants, we found K_V_7.1–L236R, K_V_7.1–W379R, and K_V_7.1–Y522S located primarily intracellularly (Fig. 7a-d). The mutant proteins seemed to be retained in the ER as illustrated by the quantification of the ratio of channels in the membrane versus channels trapped in intracellular compartments (Fig. 7e). We conclude that the loss-of-function phenotype observed in electrophysiological experiments is at least in part due to impaired trafficking.

## Discussion

In this study, we identified novel LQTS mutations in the genes *KCNH2* and *KCNQ1*. Analysis of the novel K_V_11.1 mutations R35W and S620G showed loss-of-function which could be ascribed rather to changed biophysical parameters than trafficking deficiency. For Proband-1 carrying K_V_11.1–R35W, her episodes of *torsades* storm were precipitated by hypokalemia. The role of hypokalemia as a trigger for *torsades* is well appreciated and reduced serum K^+^levels have been associated with LQTS^19^. Furthermore, low extracellular potassium levels have been shown to affect the voltage-dependence of K_V_11.1 activation and several of the kinetic parameters leading to decreased current levels^6^. Therefore, we investigated the effects of lowering potassium levels for K_V_11.1–R35W. Whereas relative reduction of the K^+^current with induced hypokalemia was similar to that found in wild-types, the absolute reduction was more pronounced, though not significant, for mutant channels.

We found that the novel mutation S620G led to a complete loss-of-function that could only be further investigated in the heterozygous state where we observed a significantly hyperpolarizing shift of the V_½_ of voltage-dependent recovery from inactivation and faster inactivation. Others have investigated channel mutants in S620 and substituted serine with other amino acids (alanine, isoleucine, phenylalanine, tyrosine, or valine) which were reported as unsuitable for analysis. We speculate that these amino acids also have a complete loss-of-function and that they might be possible to investigate in the heterozygous state – but this was not addressed in the previous studies. Substitution of S620 with cysteine or threonine almost abolished inactivation dependent on current levels. Thus, our data supports previous reports that serine 620 in K_V_11.1 is crucial for channel inactivation^11^,^12^,^13^,^14^,^20^ and voltage-dependent recovery from inactivation^11^. As K_V_11.1 mediated currents are main contributors to action potential duration in humans, changed inactivation properties may explain the phenotype of the Proband-2. The arrhythmic episodes of Proband-2 were triggered by auditory stimuli by her alarm clock. The association of auditory stimuli and impaired I_Kr_ function is well established^21^ further supporting the hypothesis that this novel mutation is disease causing.

Characterizing the novel K_V_7.1 mutations, we observed loss-of-function due to changed biophysical parameters and/or impaired trafficking that could explain the LQTS of the patients. Intriguingly, the family history of sudden death related to swimming (Probands 3, 4, and 5) supports previous studies that established swimming as a trigger for *torsades* in patients carrying mutations in the *KCNQ1* gene^22^. However, in some of the cases, family members carrying the mutations remained asymptomatic. Of note, Proband-3 (K_V_7.1–Y522S) also carried a known non-pathogenic common variant in *SCN5A (H558R)*. Conceivably, the “non-pathogenic” variant H558R in *SCN5A* may have played an adverse role in our patient.

The incidence of VUS in this cohort is higher than other cohorts. In the early days of genetic testing in cardiology nearly any variant found in a known disease gene was called pathogenic. Nowadays, with greater appreciation of the extent of rare genetic variation seen in the general population, the approach is more conservative in calling pathogenic mutations which adds to the VUS rate. Variant interpretation varies as well and the literature has many reports of “positive” mutations that are actually VUSs. We currently see lower “positive” (pathogenic) and higher VUS rates because the numbers quoted for the yield of clinical genetic testing are based on registry populations of affected families and are higher than what is seen in a typical clinical practice especially as genetic testing is extended to non-familial and/or atypical cases. Many patients in our cohort are non-familial and/or atypical cases and thus our genetic testing never approaches the yields quoted in other cohorts’ studies or by the genetic testing labs. Furthermore, a higher yield of positive results is expected since the patients were recruited from a tertiary referral center. Another reason for the difference in the VUS rate from other cohorts is that the population in San Francisco is far more ethnically diverse than other areas of the country. According to the U.S. Census Bureau, San Francisco’s current level of ethnic diversity is where the national demographic will shift to in about 30 years. The rich character and cultural composition of San Francisco make it an ideal microcosm for the U.S. population. Ethnically matched control population data is limited for non-European/non-African American patients and this also explains the difference from the incidence of VUS in our cohort from other cohorts.

## Conclusions

Of 39 probands with LQTS, six novel missense VUS were found and loss of K^+^ channel function was found in five of them. Functional characterization showed ER-retention and loss-of-function of the tested mutations indicating a strong genotype/phenotype correlation. Our study emphasizes the frequency of adverse channel function in patients with LQTS and the importance of heterologous studies to define channel function.

## Methods

### Study subjects

Over the period from 2008 to 2012 a total of 39 probands with a clinical diagnosis of long QT syndrome based on the Schwartz criteria^2^ completed clinical genetic testing for LQTS.

The study was carried out in accordance with the principles outlined in the Declaration of Helsinki and was approved by the Institutional Review Board of the University of California San Francisco. All included patients gave written informed consent.

### Mutation screening

Genetic testing was performed at either Familion (New Haven, CT, USA) or Gene Dx (Gaithersburg, MD, USA) laboratories. In all 39 probands, testing included sequence analysis of *KCNQ1*, *KCNH2*, *SCN5A*, *KCNE1*, and *KCNE2* genes. In many patients, additional LQTS genes (*ANK2*, *KCNJ2*, *CACNA1C*, *CAV3*, *SCN4B*, *AKAP9*, and *SNTA1*) were analyzed by sequencing and technologies such as multiplex ligation-dependent probe amplification that identifies deletions and duplications not detected by sequencing. Laboratory control groups consisted of 350–1300 presumably healthy subjects of Caucasian and African American ancestry depending on lab and time of testing.

### Molecular biology

The point mutations in hK_V_11.1 (GenBank Acc No. NM_000238) and hK_V_7.1 (NM_000218) were introduced using standard techniques, see Supplemental Methods for details.

### Immunofluorescence and imaging

For experimental details, see Supplemental Methods. Briefly, MDCK (strain II) cells were transfected with 3 μg of plasmid DNA using Lipofectamine and Plus Reagent (Invitrogen, Glostrup, Denmark) according to manufacturer´s protocol. Cells were stained with goat polyclonal anti-K_V_7.1 (2 μg/mL, C-20, Santa Cruz Biotechnology, Heidelberg, Germany) followed by the secondary antibody Alexa-Fluor 488 donkey anti-goat IgG (10 μg/mL, Invitrogen) The plasma membrane was visualized using rhodamine-conjugated phalloidin (1.5 U/mL, Invitrogen). Images were acquired using Zeiss LSM780 laser scanning confocal microscopy system. Quantifications were carried out using the ImageJ (version: Fiji) software, see Supplemental Methods for details.

### Two-electrode voltage-clamp electrophysiology

For experimental details, see Supplemental Methods. Briefly, *Xenopus laevis* oocytes were injected with 5 ng cRNA/oocyte for K_V_7.1 (2.5 ng + 2.5 ng upon co-expression of WT and MUT), 1 ng + 0.2 ng cRNA/oocyte for K_V_7.1 + KCNE1 (molar ratio 1:1), 1 ng cRNA/oocyte for K_V_11.1. Recordings from oocytes were performed 1-3 days after injection using a two-electrode voltage-clamp amplifier (Dagan CA-1B; Chicago, IL). Oocytes were superfused with Kulori solution (in mM: NaCl 90, KCl 4, MgCl_2_ 1, CaCl_2_ 1, HEPES 5, pH = 7.4 with NaOH). Series resistance compensation was employed. Data acquisition was performed with the Pulse software (HEKA Elektronik, Lambrecht/Pfalz, Germany). For all recordings, the holding potential was −80 mV.

### Data analysis

Data were analyzed using Igor Pro 4.04 (Wavemetrics, OR, USA) and Prism 4 (GraphPad cSoftware, CA, USA) software. Current–voltage (IV) relations were obtained from the step-protocol by plotting the outward current at the end of the test pulse as a function of the test potential. The voltage-dependence of activation was determined by fitting a Boltzmann distribution of the form I(V) = 1/(1 + exp[(V_½_ − V)/a]), where V_½_ = potential for half-maximal activation, and a = slope factor to the normalized peak tail currents. Data was normalized to the average of each batch for a given potential. Electrophysiological recordings were performed on at least three different batches of oocytes, and the number of independent experiments is indicated by n. Data are shown as mean ± SEM, and statistical significance (*p < 0.05, **p < 0.01, and ***p < 0.001) was evaluated as appropriate by two-way ANOVA followed by Bonferroni post-test or by unpaired Student’s t-test.

## Limitations

Genetic screening at commercial providers included the most frequent LQTS genes and we cannot exclude mutations in other or yet unknown genes. Also, not all family members were available for genetic testing. We limited our functional analysis to variants in the protein encoding region. We chose the *Xenopus laevis* expression system as it allows for controlled expression levels and subunit ratios due to direct injection of precise cRNA amounts and for fast functional screening of a number of mutations. Yet, this conventional heterologous expression system differs from that in native cardiomyocytes and lower temperature may rescue otherwise trafficking deficient mutants.

## Additional Information

**How to cite this article**: Steffensen, A. B. *et al*. High incidence of functional ion-channel abnormalities in a consecutive Long QT cohort with novel missense genetic variants of unknown significance. *Sci. Rep.* **5**, 10009; doi: 10.1038/srep10009 (2015).

## Supplementary Material

Supplementary Information
