# Supplementary material for: High incidence of functional ion-channel abnormalities in a consecutive Long QT cohort with novel missense genetic variants of unknown significance
Source: Sci Rep. 2015 Jun 12;5:10009. doi: 10.1038/srep10009 (PMC4464365; doi:10.1038/srep10009)
Supplement: Supplementary Information [file srep10009-s1.doc]

**SUPPLEMENTAL MATERIAL**

**High incidence of functional ion-channel abnormalities in a consecutive Long QT cohort with novel missense genetic variants of unknown significance**

Short title: Steffensen, Refaat, Novel LQTS mutations

Annette Buur Steffensen^1,6#^, Marwan M. Refaat^2#^, Jens-Peter David^1^, Amer Mujezinovic^1^, Kirstine Calloe^1,5^, Julianne Wojciak^3,4^, Robert L. Nussbaum^3,4^, Melvin M. Scheinman^3,4^, Nicole Schmitt^1*^

^1^Danish National Research Foundation Centre for Cardiac Arrhythmia, Department of Biomedical Sciences, University of Copenhagen, Copenhagen, Denmark
^2^Department of Internal Medicine, Division of Cardiology, American University of Beirut Medical Center, Beirut, Lebanon
^3^Department of Medicine and ^4^Institute for Human Genetics, University of California San Francisco, California, U.S.A.

^5^Present address: Department of Veterinary Clinical and Animal Science, University of Copenhagen, Copenhagen, Denmark

^6^Present address: Department of Cellular and Molecular Medicine, Faculty of Health and Medical Sciences, University of Copenhagen, Denmark

#equal contribution

**Content**

[SUPPLEMENTAL METHODS 3](#__RefHeading___Toc411928583)

[Molecular biology 3](#__RefHeading___Toc411928584)

[Immunofluorescence and imaging 3](#__RefHeading___Toc411928585)

[Quantification of surface expression 4](#__RefHeading___Toc411928586)

[Two-electrode voltage-clamp electrophysiology 4](#__RefHeading___Toc411928587)

[SUPPLEMENTAL TABLES AND FIGURES 5](#__RefHeading___Toc411928588)

[Supplemental Table S1: Genetic variants identified in LQTS patients 5](#__RefHeading___Toc411928589)

[Supplemental Figure S1. Pedigree of proband carrying K_V_11.1 R35W 8](#__RefHeading___Toc411928590)

[Supplemental Figure S2. Helical net presentation of S4 for WT (left) and mutant L236R 9](#__RefHeading___Toc411928591)

[Supplemental Figure S3. Pedigree of proband carrying K_V_7.1 W379R and K_V_11.1 V491I. 10](#__RefHeading___Toc411928592)

[Supplemental Figure S4. Positions of the identified mutations and polymorphism 11](#__RefHeading___Toc411928593)

[Supplemental Figure S5. Characterization of K_V_11.1-R35W. 12](#__RefHeading___Toc411928594)

[Supplemental Figure S6. Characterization of K_V_7.1-W379R. 14](#__RefHeading___Toc411928595)

[SUPPLEMENTAL REFERENCES 16](#__RefHeading___Toc411928596)

# SUPPLEMENTAL METHODS

## Molecular biology

The point mutations R35W (c.103C>T), V491I (c.1471G>A), and S620G (c.1858A>G) in K_V_11.1, and L236R (c.707T>G), W379R (c.1135T>C), and Y522S (c.1565A>C) in K_V_7.1 were introduced using mutated oligonucleotide extension (PfuTurbo Polymerase, Stratagene, La Jolla, CA, USA) from the plasmid template harboring the cDNA of interest, digested with DpnI (Fermentas, St. Leon-Roth, Germany) and transformed into *E.coli* XL1 Blue cells. All plasmids were verified by complete DNA sequencing of the cDNA insert (Macrogen Inc., Seoul, Rep. of Korea). cRNA was prepared from linearized plasmids comprising hK_V_7.1 (GenBank Acc No. NM_000218), hKCNE1 (NM_000219), or hK_V_11.1 (NM_000238) using the mMESSAGE mMACHINE T7 kit (Ambion, Nærum, Denmark).

## Immunofluorescence and imaging

For imaging, MDCK (strain II) cells were grown in T75 flasks (Nunc, Roskilde, Denmark) in Dulbecco's modified Eagle's medium (DMEM) (in-house, Faculty of Health and Medical Sciences) supplemented with 100 U/mL penicillin, 100 mg/mL streptomycin (Invitrogen, Glostrup, Denmark) and 10% fetal calf serum (FCS) (Sigma-Aldrich, Copenhagen, Denmark) at 37°C in 5% CO_2_ until confluence. Cells were transfected with 3 µg of plasmid DNA using Lipofectamine and Plus Reagent (Invitrogen, Glostrup, Denmark) according to manufacturer´s protocol. Cells were plated on glass cover slips (12 mm in diameter, Thermo Fischer Scientific, Roskilde, Denmark) and incubated 48-72 hours at 37°C in 5% CO_2_.

Transfected cells were fixed in 4% paraformaldehyde in phosphate buffered saline (PBS) for 30 minutes at room temperature. Cells were blocked and permeabilized in 0.1% Triton X-100/0.2% fish skin gelatin in PBS (immunobuffer, IB) for 30 minutes prior to one hour incubation with goat polyclonal anti-K_V_7.1 (2 μg/mL, C-20, Santa Cruz Biotechnology, Heidelberg, Germany, in IB). Followed by three washing steps Alexa-Fluor 488 donkey anti-goat IgG (10 μg/mL, Invitrogen) diluted in IB was applied for 45 minutes. The plasma membrane was visualized using rhodamine-conjugated phalloidin (1.5 U/mL, Invitrogen). Cover slips were mounted in Prolong Gold Antifade reagent (Invitrogen) and images were acquired using Zeiss LSM780 laser scanning confocal microscopy system with a 63x/numerical aperture (NA) = 1.40 oil objective and the pinhole diameter was set between 0.9-1.0 µm. Line averaging and sequential scanning was applied to reduce noise and allow separation of the individual channels, respectively. All images were obtained with a pixel format of 1024x1024 and treated using ZEN 2010 Edition and Illustrator CS5.

## Quantification of surface expression

The relative surface expression of K_V_7.1, K_V_7.1-L236R, K_V_7.1-W379R, K_V_7.1-Y522, K_V_11.1, K_V_11.1-R35W, and K_V_11.1-S620G was determined using the ImageJ (version: Fiji) software. A 10 pixel wide line was drawn from outside the cell into the cell (avoiding the nucleus) at places where phalloidin staining (used as a membrane marker) was well visualized. For the length of that line the intensity of the fluorescence signal from both the phalloidin and from each of the above mentioned channels were measured. First, the point on the line with the highest intensity of phalloidin signal was found. At that point the intensity of the signal from channel staining was used as a measurement of channels in the membrane. Next, as a measurement of channels inside the cell, the intensity of channel fluorescence signals at a point 0,75 μm further into the cell was used. The ratio between these two measurements was calculated and the mean value of three ratios from each cell was reported. 18-29 cells from three (K_V_11.1) or four (K_V_7.1) independent experiments for each situation was used for statistical analysis. A one-way ANOVA followed by a Tukey´s range test was performed in Prism5. The data are presented as mean ± SEM and P=0.05 was considered significant.

## Two-electrode voltage-clamp electrophysiology

*Xenopus laevis* oocytes were purchased from EcoCyte Bioscience (Castrop-Rauxel, Germany). 50 nl cRNA was injected using a Nanoject microinjector (Drummond Scientific, Broomall, PA), 5 ng cRNA/oocyte for K_V_7.1 (2.5 ng+2.5 ng upon co-expression of WT and MUT), 1 ng+0.2 ng cRNA/oocyte for K_V_7.1+KCNE1 (molar ratio 1:1), 1 ng cRNA/oocyte for K_V_11.1. Oocytes were kept at 19°C and currents were measured 1-3 days after injection.

Recordings from oocytes were performed using a two-electrode voltage-clamp amplifier (Dagan CA-1B; Chicago, IL). Borosilicate glass recording electrodes (Module Ohm, Herlev, Denmark) were made using a DMZ-Universal Puller (Zeitz Instruments, Germany) and had a resistance of 0.5 to 1 MΩ when filled with 2 M KCl. Oocytes were superfused with Kulori solution (in mM: NaCl 90, KCl 4, MgCl_2_ 1, CaCl_2_ 1, HEPES 5, pH = 7.4 with NaOH) and experiments were performed at room temperature. Data acquisition was performed with the Pulse software (HEKA Elektronik, Lambrecht/Pfalz, Germany). For all recordings, the holding potential was -80 mV.

# SUPPLEMENTAL TABLES AND FIGURES

## Supplemental Table S1: Genetic variants identified in LQTS patients

| **No.** | **Variation in** | | | **Comments** | **PolyPhen-2 Prediction** | **Mutation Taster** | **SIFT** | **Proband No. (this study)** |
| --- | --- | --- | --- | --- | --- | --- | --- | --- |
|  | **K_V_7.1**  **(LQT1, *KCNQ1*)** | **K_V_11.1**  **(LQT2, *KCNH2*)** | **Na_V_1.5**  **(LQT3, *SCN5A*)** |  |  |  |  |  |
| **1** |  | R100Q^a,1^ |  |  |  |  |  |  |
| **2** | G350R^a,1^ |  |  |  |  |  |  |  |
| **3** |  |  |  | *KCNE1*K70M^a,1^ |  |  |  |  |
| **4** | Q531X^c^ | D323N^b^ |  |  |  |  |  |  |
| **5** | A150T^b^ |  |  |  |  |  |  |  |
| **6** | G168R^a,1^ |  |  |  |  |  |  |  |
| **7** | IVS8+5G>A^b,*^ |  |  |  |  |  |  |  |
| **8** |  |  | N406K^a,1^ |  |  |  |  |  |
| **9** | **W379R^c,*^** | **V491I^b,*^** | P2005L^b^ | *SCN5A* P2005A reported^2^ | W379R; Probably Damaging  V491I; Benign | W379R; Disease causing  V491I; Polymorphism | W379R; Damaging  V491I; Tolerated | **5** |
| **10** | G269S^a,1^ |  |  |  |  |  |  |  |
| **11** |  | Q376Q^a,1^ |  |  |  |  |  |  |
| **12** |  |  |  | None detected |  |  |  |  |
| **13** | V254M^a,1^ |  |  |  |  |  |  |  |
| **14** |  | L602P^c^ |  |  |  |  |  |  |
| **15** | del ex7-10^a,1^ |  |  |  |  |  |  |  |
| **16** |  |  |  | None detected |  |  |  |  |
| **17** |  | F98S^a,1^ |  |  |  |  |  |  |
| **18** |  | **R35W^c,*^** |  |  | Damaging | Disease causing | Damaging | **1** |
| **19** | **L236R^b,*^** |  |  |  | Probably Damaging | Disease causing | Damaging | **4** |
| **20** |  |  |  | None detected |  |  |  |  |
| **21** |  |  |  | None detected |  |  |  |  |
| **22** |  |  |  | None detected |  |  |  |  |
| **23** |  | Y493X^a,1^ |  |  |  |  |  |  |
| **24** |  |  |  | *AKAP9* T3473NfsX3^b^  *CACNA1C* T1787M^d, 3^ |  |  |  |  |
| **25** | R190Q^a,1^ | P1093L^c^ |  |  |  |  |  |  |
| **26** |  |  |  | None detected |  |  |  |  |
| **27** |  |  |  | None detected |  |  |  |  |
| **28** |  | delEx12-14^a,1^ |  |  |  |  |  |  |
| **29** | W323X^a^ |  |  |  |  |  |  |  |
| **30** |  |  |  | None detected |  |  |  |  |
| **31** |  | **S620G^c,*^** |  |  | Benign | Disease causing | Damaging | **2** |
| **32** |  |  |  | None detected |  |  |  |  |
| **33** |  | L552S^a,1^ |  |  |  |  |  |  |
| **34** |  |  |  | None detected |  |  |  |  |
| **35** |  |  |  | None detected |  |  |  |  |
| **36** | P448QfsX18^a,1^ |  |  | *ANK2* T492A^b^ |  |  |  |  |
| **37** | K218E^a,1^ | G1036D^c^ |  | *KCNH2* G1036+82X reported^4^ |  |  |  |  |
| **38** |  | G572S^c^ |  |  |  |  |  |  |
| **39** | **Y522S^c,*^** |  |  |  | Possibly Damaging | Disease causing | Damaging | **3** |

Gene names are indicated in *italics*. ^a^reported pathogenic; ^b^variant with unknown significance (VUS); ^c^VUS likely pathogenic; ^d^VUS likely benign; *novel VUS at time of inclusion in this study; del, deletion; ex, exon


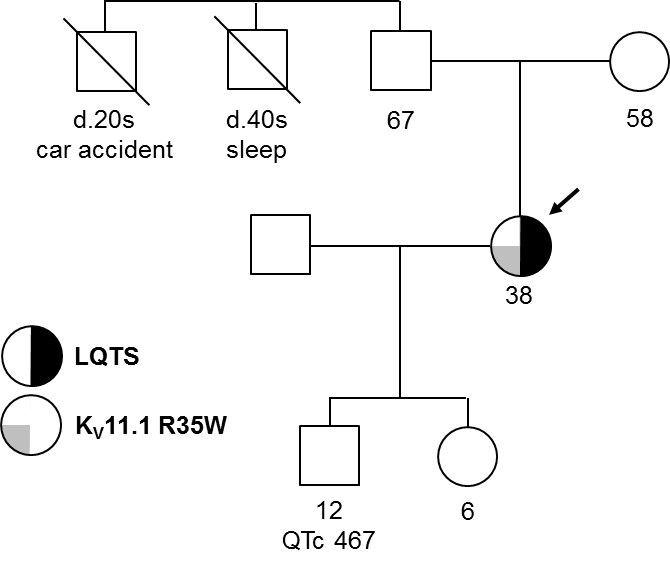


Supplemental Figure S1. Pedigree of proband carrying K_V_11.1 R35W (circles = females; squares = males) with phenotypic and genotypic information where available. Age at diagnosis/inclusion given where available. QTc interval in ms. Parents were not tested. The arrow marks Proband-1.

**
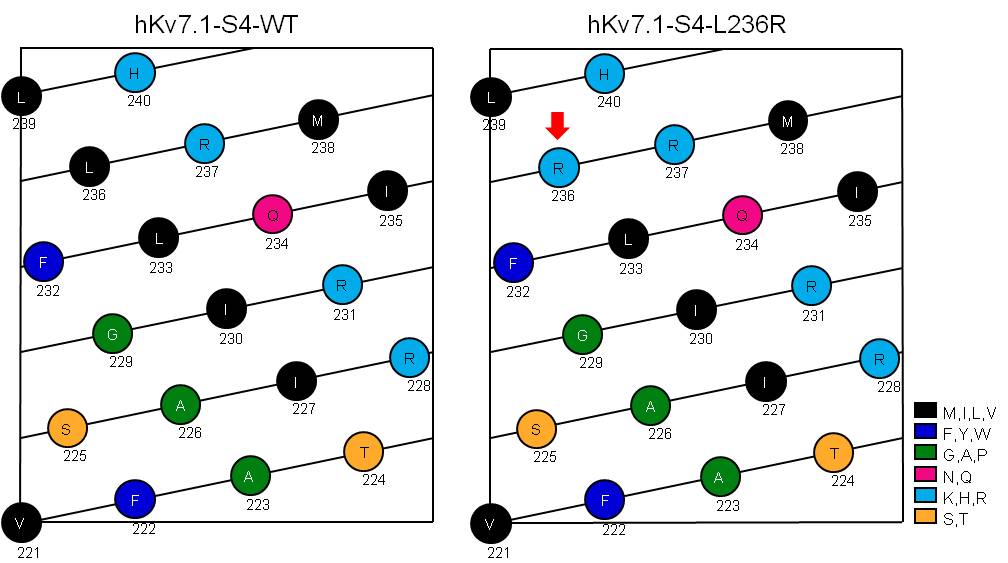
**

Supplemental Figure S2. Helical net presentation of S4 for WT (left) and mutant L236R (right) channel showing the amino acids arranged on a cylindrical helix that is cut open and unfolded, running from the amino terminus on the bottom to the carboxyl terminus on top. The residue scale indicates which group each amino acid belongs to. The red arrow denotes the position of the mutation identified in Proband-4. Analysis was performed using the Protean software (Lasergene DNASTAR, Madison, USA).

**
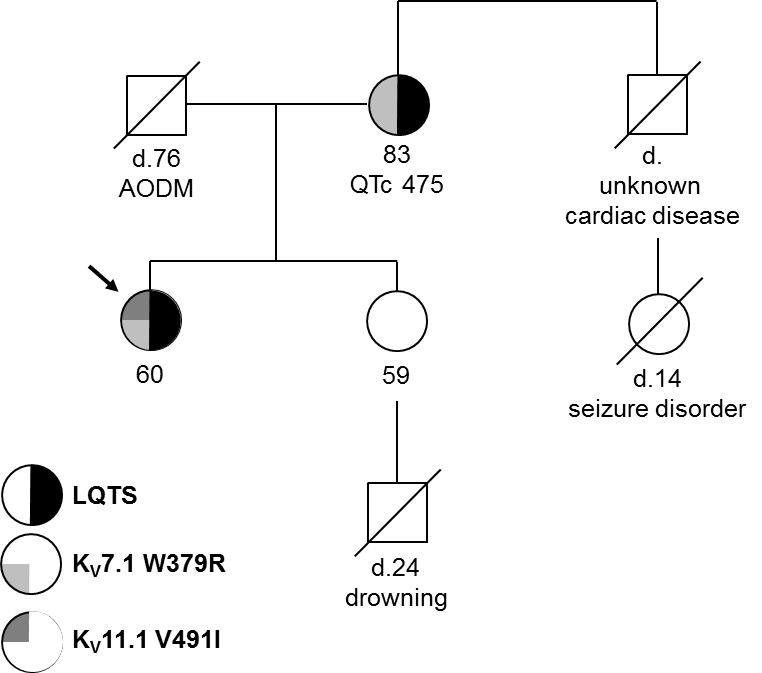
**

Supplemental Figure S3. Pedigree of proband carrying K_V_7.1 W379R and K_V_11.1 V491I. Symbols as in Supplemental Figure S1.

**
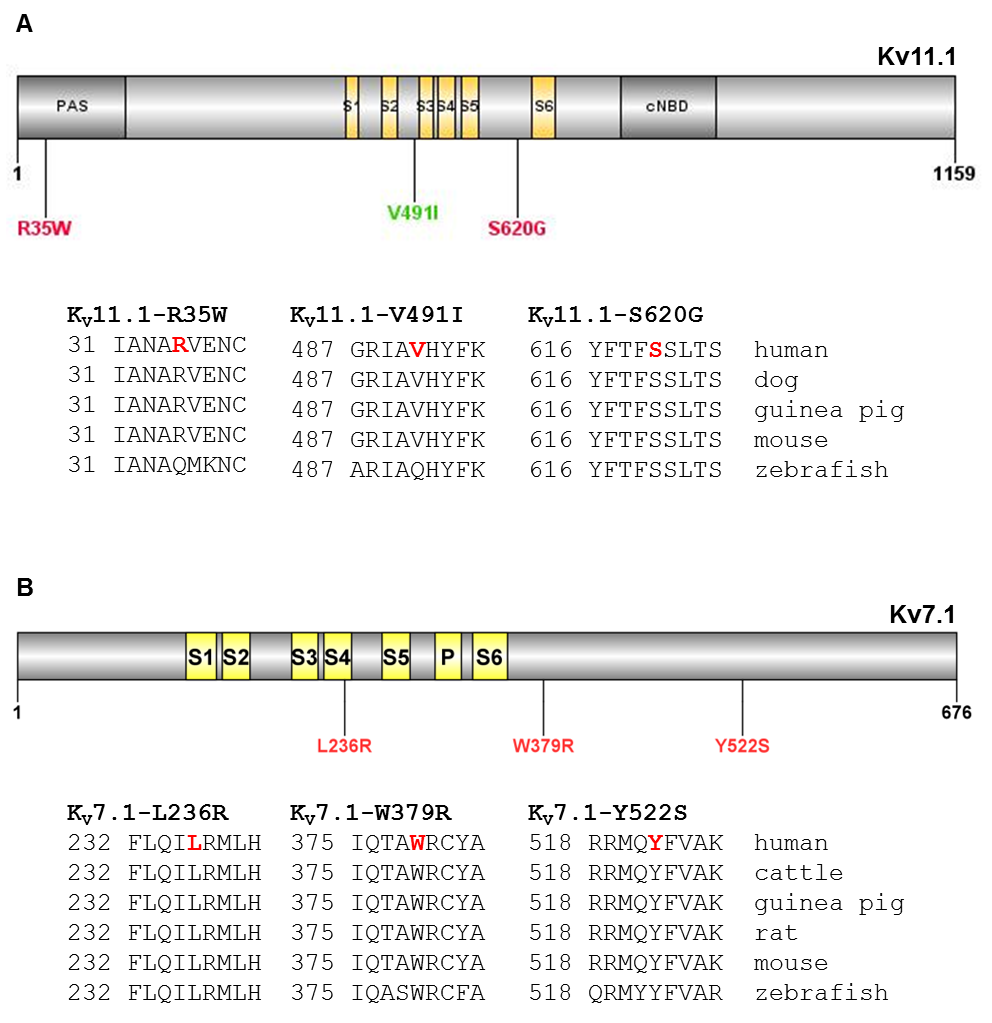
**

Supplemental Figure S4. Positions of the identified mutations and polymorphism (illustrated with DOG 1.0 software ^5^) (**A**) Topological depiction of K_V_11.1 that comprises so-called PAS domain at its distal N-terminus and a cyclic nucleotide binding domain (cNBD) in its C-terminus (upper panel), S1-S6 transmembrane segments, P pore. In red loss-of-function mutations, in green benign variants or gain-of-function mutations. Amino acid alignment of K_V_11.1 protein sequences (lower panel) from human (GenBank Acc.No NP_000229), dog (NP_001003145), guinea pig (NP_001166444), mouse (NP_038597), and zebrafish (NP_998002). (**B**) Topological depiction and of amino acid alignment of K_V_7.1 sequences from human (GenBank Acc.No NP_000209), cattle (NP_001192370), rat (NP_114462), guinea pig (NP_001166292), and zebrafish (NP_001116714). Labeling as in (**A**).

Supplemental Figure S5. Characterization of K_V_11.1-R35W. WT or KV11.1-R35W was expressed in Xenopus laevis oocytes and currents were recorded in Kulori’s solution containing either 1 or 4 mM KCl. A: Representative current traces. B: I/V relationship for KV11.1. Values at 0 mV; WT 4 mM KCl 2.7±0.2 µA, WT 1 mM KCl 1.2±0.1 µA, and KV11.1-R35W 4 mM KCl; 1.7±0.1 µA, KV11.1-R35W 1 mM KCl; 1.0±0.1 µA. Asterisks indicate statistically significant differences for 4 mM compared with 1 mM for either KV11.1 or KV11.1-R35W. C: Normalized peak tail current. Boltzmann functions were fit to the data. For KV11.1, the half-maximal activation voltage (V1/2) was -12.0±1.3 mV (at 4 mM KCl) or -8.5±1.7 mV (1 mM KCl) (n = 5); for K_V_11.1-R35W: V_1/2_ = -11.2±1.1 mV (4 mM KCl) and -9.5±1.0 mV (1 mM KCl) (n = 8). *P<0.05, **P<0.01, ***P<0.001.

Supplemental Figure S6. Characterization of K_V_7.1-W379R. **A**: Representative current traces recorded from *X. laevis* oocytes injected with K_V_7.1 or K_V_7.1-W379R cRNA; both with KCNE1 in a 1:1 molar ratio. The current step protocol is shown as inset. **B**: Currents measured at the end of each step were used to construct the current-voltage (I/V) relationship for K_V_7.1 (at 40 mV; 7.1±0.7 µA, n = 15), K_V_7.1+K_V_7.1-W379R (at 40 mV; 1.7±0.2 µA, n = 15), and K_V_7.1-W379R (at 40 mV; 0.5±0.1 µA, n = 15). **C**: Voltage-dependent activation of the channels was determined from the normalized peak tail currents by fitting Boltzmann functions. For K_V_7.1 the half-maximal activation voltage (V_1/2_) was 27.7±1.4 mV, K_V_7.1+K_V_7.1-W379R: V_1/2_ = 39.8±2.4 mV. For K_V_7.1-W379R the tail current was so small that V_½_ could not be assessed. **D**: The I/V relationship for KCNE1 alone (at 40 mV; 0.9±0.1 µA, n = 15) and empty oocytes (at 40 mV; 0.1±0.03 µA, n = 9). **E**: Voltage-dependent activation of the KCNE1 alone and empty oocytes was determined without normalization due to very small current levels. For KCNE1 the half-maximal activation voltage (V_1/2_) was 28.3±1.8 mV, and empty oocytes: V_1/2_ = -46.8±5.5 mV. **P<0.01, ***P<0.001.

# SUPPLEMENTAL REFERENCES

1. Napolitano,C. & Wilson J. Gene Connection of the Heart [Internet]. 2010, Available

from: http://www.fsm.it/cardmoc/

2. Shim,S.H., Ito,M., Maher,T., & Milunsky,A. Gene sequencing in neonates and infants with the long QT syndrome. *Genetic. testing.* **9**, 281-284 (2005).

3. Burashnikov,E. *et al.* Mutations in the cardiac L-type calcium channel associated with inherited J-wave syndromes and sudden cardiac death. *Heart Rhythm.* **7**, 1872-1882 (2010).

4. Berthet,M. *et al.* C-terminal HERG mutations the role of hypokalemia and a KCNQ1-associated mutation in cardiac event occurrence. *Circulation* **99**, 1464-1470 (1999).

5. Ren,J. *et al.* DOG 1.0: illustrator of protein domain structures. *Cell research* **19**, 271-273 (2009).
